# Hip fractures: public perceptions are we jumping the gun?

**DOI:** 10.1308/003588412X13373405386051

**Published:** 2012-09

**Authors:** N Weston, S Lambert, CWM Horner, T McBride

**Affiliations:** The Royal Wolverhampton Hospitals NHS Trust

## Abstract

**McBride TJ, Panrucker S, Clothier JC** Hip fractures: public perceptions. *Ann R Coll Surg Engl* 2011; **93**: 67–70 doi: 10.1308/003588411X12851639107034

We read with interest the article by McBride *et al*, which reported that only 4% of 127 patients and relatives in an outpatient clinic post hip replacement following a hip fracture correctly acknowledged one-year mortality for hip fractures to be approximately 30%. Poor knowledge retention of doctor–patient consultations (20-60%)^1^ does not explain this figure fully.

To investigate further we undertook a retrospective review of case notes for patients presenting with hip fractures during the period July to August 2011, searching for evidence that patients and their relatives were being given information on morbidity and mortality. Written documentation was only found on 10 occasions (28.6%). Although the risks and benefits of surgery are discussed with the patient, it would seem that the severity of the injury itself is not communicated adequately.This might explain why the public grossly underestimates the mortality of sustaining a hip fracture.

Discussing such sensitive issues in the acute setting can be very distressing for both the patient and the doctor but it is important to discuss the diagnosis itself to avoid creating unfair and unrealistic expectations of treatment. Perhaps an information leaflet should be incorporated into current hip fracture pathways?
